# Isolation of *Vibrio cholerae* El Tor Serotype Inaba in 2006 and the Most Common Phage Type of *V. cholerae* in Mumbai

**DOI:** 10.4103/0970-0218.45385

**Published:** 2009-01

**Authors:** Anuradha De, Meenakshi Mathur

**Affiliations:** Department of Microbiology, L.T.M. Medical College, Sion, Mumbai - 400 022, Maharashtra, India. E-mail: dr.anuradhade@yahoo.com

Sir,

A total of 168 strains of *Vibrio cholerae* were isolated and tested over a period of 3 years (2004-2006). The strains were identified by standard methods(1) and were identified using a slide agglutination test with *Vibrio cholerae* O1 antisera and biotyped using a polymyxin B sensitivity test. The non-agglutinating strains were tested with *V. cholerae* O-139 antisera. Of 168 strains, 96 were isolated in 2004, 39 were isolated in 2005, and 33 were isolated in 2006.

Eighty-three *V. cholerae* O1 isolates were sent to the National Institute of Cholera and Enteric Diseases (NICED) in Kolkata for serotyping and phagetyping. All the *V. cholerae* O1 isolates were of El Tor biotype. Among the two non-agglutinating vibrios isolated in 2005, both did not agglutinate with O-139 antisera. *V. cholerae* El Tor serotype Inaba was found only in 2006. The isolates of 2004 and 2005 were of the Ogawa serotype. In a previous study in the same institute, all isolates detected over a period of 5 years (1996-2000) were of the Ogawa serotype.(2) From 2001 to 2005, all isolates were *V. cholerae* El Tor Ogawa (unpublished). We had isolated serotype Inaba in 2006 for the first time. A shift in the occurrence of Ogawa and Inaba serotypes in a given area are thought to be a consequence of the genetic reversal that occurs *in-vivo* and *in-vitro* and is possibly mediated by the immune pressure in the population.(3) It appears that as an alternate to the Ogawa serotype, Inaba have appeared to aid the persistence of cholera and thus perpetuate the spread of *Vibrio cholerae* El Tor.

All the isolates during 2004 to 2006 were Basu and Mukherjee phage type 2. Turbadkar *et al*.(4) from Mumbai have reported Ogawa serotypes in 2004 and all belonged to phage type 4.

In the new phage typing scheme, out of 26 isolates sent to NICED in 2004, 14 were T26 and 12 were T27. Out of 39 isolates sent in 2005, 33 were T27 and 6 were other phage types (T13, T21, T22, T25, T23, or T15). In 2006, out of 18 isolates sent to NICED, 17 were T27 and only 1 was T26. Therefore, the most common phage type in this part of Mumbai is T27 (74.7%), followed by T26 (18.1%). In the study by Turbadkar *et al*.,(4) the majority belonged to phage type 27 (97.5%), which is in accordance with the present study.

The 168 isolates of *V.cholerae* showed maximum sensitivity to amikacin (92.3%), followed by cefotaxime (89.9%). Tetracycline sensitivity was 91.1% followed by norfloxacin (86.3%). In the previous study during 1996-2000, tetracycline sensitivity was 39.6% and norfloxacin sensitivity was 46.2%.(2) Therefore, tetracycline and norfloxacin sensitivity have increased over the years. Co-trimoxazole and nalidixic acid susceptibility was only 2.4% and 1.2%, respectively in the present study. A decrease in sensitivity to nalidixic acid was observed in the present study (13.6% in previous study).(2)

## References

[1] Cruickshank R, Duguid JP, Marmion BP, Swain RH (1985). Vibrio: Spirillum. Medical Microbiology.

[2] Mathur M, De A, Saraswathi K, Varaiya A, Athalye S (2003). Vibrionaceae from cases of acute diarrhoea and their antimicrobial sensitivity pattern: A five year prospective study. Indian J Med Microbiol.

[3] Bajaj JK, Baradkar VP, Joshi SG, Damle AS, Kayararte RP, Deshmukh AB (2001). Epidemiology of Cholera: A five year study. J Commun Dis.

[4] Turbadkar SD, Ghadge DP, Patil S, Chowdhary AS, Bharadwaj R (2007). Circulating phage type of Vibrio cholerae in Mumbai. Indian J Med Microbiol.

